# Hydrogel-forming microarray patch mediated transdermal delivery of tetracycline hydrochloride

**DOI:** 10.1016/j.jconrel.2023.02.031

**Published:** 2023-03-07

**Authors:** Li Zhao, Lalitkumar K. Vora, Stephen A. Kelly, Linlin Li, Eneko Larrañeta, Helen O. McCarthy, Ryan F. Donnelly

**Affiliations:** School of Pharmacy, Queen’s University Belfast, 97 Lisburn Road, Belfast BT9 7BL, United Kingdom

**Keywords:** Antibiotic resistance, Transdermal, Microarray patch, Tetracycline hydrochloride

## Abstract

Antibiotic resistance is one of the most serious health problems today and is expected to worsen in the coming decades. It has been suggested that antibiotic administration routes that bypass the human gut could potentially tackle this problem. In this work, an antibiotic hydrogel-forming microarray patch (HF-MAP) system, which can be used as an alternative antibiotic delivery technology, has been fabricated. Specifically, poly(vinyl alcohol)/poly(vinylpyrrolidone) (PVA/PVP) microarray showed excellent swelling properties with >600% swelling in PBS over 24 h. The tips on the HF-MAP were proven to be able to penetrate a skin model which is thicker than *stratum corneum*. The antibiotic (tetracycline hydrochloride) drug reservoir was mechanically robust and dissolved completely in an aqueous medium within a few minutes. *In vivo* animal studies using a Sprague Dawley rat model showed antibiotic administration using HF-MAP achieved a sustained release profile, in comparison with animals receiving oral gavage and intravenous (IV) injection, with a transdermal bioavailability of 19.1% and an oral bioavailability of 33.5%. The maximum drug plasma concentration for HF-MAP group reached 7.40 ± 4.74 μg/mL at 24 h, whereas the drug plasma concentration for both oral (5.86 ± 1.48 μg/mL) and IV (8.86 ± 4.19 μg/mL) groups peaked soon after drug administration and had decreased to below the limit of detection at 24 h. The results demonstrated that antibiotics can be delivered by HF-MAP in a sustained manner.

## 1 Introduction

The discovery and clinical application of antibiotics has been a major breakthrough in the history of modern medicine. Antibiotics continue to play an important role in treating infections and ensuring safety during various medical procedures [1]. However, antibiotic resistance can be dramatically accelerated by the misuse of antibiotics. According to WHO, antibiotic resistance has become one of the ten biggest threats to health today [2]. It is expected that, by 2050, antibiotic resistance will overtake cancer as the biggest health threat for humanity, killing an extra 10 million people every year [3,4]. For a long time, the problem of antibiotic resistance has not received enough social and political interest and insufficient efforts have been made to develop new antibiotics [5]. Moreover, pharmaceutical companies are reluctant to develop new antibiotics due to their relatively low profitability in comparison with other drug types, such as anti-cancer therapeutics [6]. Accordingly, there have not been any new classes of antibiotics developed in recent decades [7,8]. As a result, it is of great importance to extend the lifespan of the currently available antibiotics, which could save many lives and reduce associated social and economic costs.

Previous research has shown that oral administration of antibiotics could significantly accelerate the development of antibiotic resistance because antibiotics interact with bacteria inhabiting the human gut [9]. The overexposure of the gut microbiota to antibiotics can cause various problems such as dysbiosis and increased susceptibility to infection. Zhang *et al* confirmed that IV injection of renally excreted antibiotics could considerably slow down the development of antibiotic resistance among gut bacteria relative to oral administration of the same drugs [10]. However, it is impractical for patients to inject themselves with antibiotics at home. IV injection of antibiotics in hospital results in much higher costs than at-home oral administration, causing a heavy burden on finance and resources for healthcare providers. Accordingly, an alternative antibiotic administration route which can bypass the human gut, while allowing patients to be able to use it independently is highly desirable.

The minimally invasive microarray patch (MAP) technology has been investigated as an important alternative transdermal drug delivery technology because it can deliver drugs systemically with self-administration [11–15]. The small and short projections on the MAP can painlessly penetrate the *stratum corneum* before delivering the drugs into the epidermis. Drug molecules will then be absorbed by the rich microcirculation within the dermis. There are a variety of different types of MAP technologies, such as solid, coated, dissolving, and hollow microneedle (MN) technologies [16–20]. However, only hydrogel-forming MAP (HF-MAP) has successfully been used to delivered high dose hydrophilic compounds transdermally, bypassing the *stratum corneum* [21–25]. A HF-MAP system consists of a HF-MAP as well as a solid drug reservoir which can be attached to the back of the HF-MAP [26–30]. Upon insertion into the skin, the interstitial fluid uptaken by the HF-MAP can dissolve the drug reservoir and the dissolved drug will then permeate into skin. The size of the drug reservoir can be tailored flexibly to meet the required drug dosages, which allows a large amount of hydrophilic compound be possibly delivered into skin [31].

In this work, we are developing a high dose tetracycline hydrochloride HF-MAP system, which allows tetracycline to be delivered into the rich dermal microcirculation in the skin, thus possibly bypassing the gut bacteria. Tetracycline is selected as the drug of interest because it is a widely used antibiotic to treat both Gram-positive and Gram-negative infections.

## 2 Materials and methods

### 2.1 Materials

Acetonitrile, citric acid, triethylamine, phosphate-buffered saline (PBS), potassium phosphate monobasic, and poly(vinyl alcohol) (PVA) (Mw = 85–124 kDa) 87–89% hydrolyzed were all purchased from Sigma-Aldrich, Steinheim, Germany. Croscarmellose sodium (CCS), marketed as *ac*-di-sol was acquired from FMC Health and Nutrition Ltd. (Philadelphia, USA). Poly(vinylpyrrolidone) (PVP) K29–32 (Mw = 58 kDa) was kindly provided by Ashland, Kidderminster, UK. Tetracycline hydrochloride (TC HCL) was kindly donated by Suzhou Zhuoqun Pharmaceutical Technology Ltd., Suzhou, Jiangsu Province, China. PEARLITOL® in the form of Pearlitol 50C, and phosphoric acid were purchased from Roquette (Lestrem, France) and Fluorochem Ltd. (Hadfield, UK), respectively. Stillborn piglets were sourced from local farms in Northern Ireland and were stored in freezer at –20 °C before further use. Parafilm® was purchased from Bemis Company, Inc.

### 2.2 Preparation of PVP/PVA HF-MAP

The PVP/PVA HF-MAP has previously been developed in our research group [29]. In this work, the same procedure was used to fabricate these HF-MAPs. To prepare PVP/PVA HF-MAPs, an aqueous mixture of PVA, PVP and citric acid was firstly obtained which contained 10% *w*/w of PVP, 15% w/w of PVA, 1.5% w/w of citric acid, with the rest being water. Afterwards, the mixture was poured into the silicone moulds, which were then centrifuged (Eppendorf Centrifuge 5804, Hamburg, Germany) at 3500 rpm for 15 min to allow the gel mixture to fill the MN tips in the moulds, as well as to remove any air bubbles in the mixture. The gel filled moulds were then left at room temperature for 48 h to allow water content to fully evaporate, before being placed in an oven (Gallenkamp Hotbox Oven Size 2, Cambridge, UK) for esterification-based crosslinking (between PVA and citric acid) at 130 °C for 3 h. The resulting PVP/PVA MAPs (11 × 11 microneedle density, 600 μm microneedle height, 300 μm width at base and 150 μm interspacing) were always stored in a desiccator to prevent them from absorbing moisture from the environment.

### 2.3 Swelling studies for PVP/PVA HF-MAP

The mass of six replicates of PVP/PVA HF-MAPs (M0) were first weighed using a balance (Kern ABJ-NM/ABS-N, Balingen, Germany). They were individually immersed in a weighting boat containing 20 mL of phosphate-buffered saline (PBS) buffer solution (pH 7.4) to allow the HF-MAP to fully swell. Their swelling was measured at 0.5, 1, 2, 3, 4, 6, and 24 h. At each time point, the HF-MAP was removed from the PBS buffer solution and a piece of laboratory tissue paper was used to absorb the fluid on the surface of HF-MAP before measuring their masses (Mt). The percentage of swelling is calculated using Eq. (1):

(1) $$Swelling\;(\%)=\frac{\text{Mt}-\text{M}0}{\text{M}0}\times 100$$

### 2.4 Mechanical and penetration tests for PVP/PVA HF-MAPs

The processes of mechanical and penetration tests have previously been reported [31]. A Texture Analyser (TA, StableMicroSystem, Surrey, UK) was used to perform the height reduction and penetration tests for the PVP/PVA HF-MAPs. Typically, a HF-MAP was first attached to the moving probe of the TA using double-sided sticky tape. After setting up the parameters on the controlling software (a test speed of 1.19 mm/s, a force of 32 N lasting for 30 s), the HF-MAP attached testing probe would automatically move towards a square aluminium block and against it once in contact. For Parafilm penetration test, an 8-layered Parafilm model was placed on the aluminium block. The Parafilm inserted by HF-MAP was first observed using optical coherence tomography (Michelson Diagnostic, Maidstone, UK). Then this Parafilm as well as the resulting compressed PVP/PVA HF-MAPs were both observed under digital microscope (Leica EZ4W, Leica, Wetzlar, Germany). The height reduction can be calculated from the MN heights before and after mechanical tests. Parafilm penetration was inspected visually under the microscope. The holes generated due to MN insertions were counted for each Parafilm layer. Eq. (2) was used to calculate penetration efficiency.

(2) $$\text{Penetration}\;\text{efficiency}\;(\%)=\frac{\text{number}\;\text{of}\;\text{holes}}{121}\times 100$$

### 2.5 Preparation of tetracycline drug tablets

To prepare tetracycline hydrochloride drug tablets, the drug powder and the excipients, which include mannitol and croscarmellose sodium, were first homogenised using mortar and pestle. The homogenised powder mixture was then aliquoted into 2 ml Eppendorf tubes. Direct compression was used to make the solid drug tablets. Typically, the aliquoted drug/excipient mixture was poured into a metal die, which was placed within a hydraulic press (Specac™ Atlas™ Manual 15 T Hydraulic Press, Specac, Orpington, UK). One tonne of force was used for compression which lasted for 30 s. The resulting drug tablets were always stored in a desiccator before use to prevent moisture absorption.

### 2.6 Physical characterisation of directly compressed tetracycline drug tablets

The break force of directly compressed tablets (DCTs) was measured using a fracturability test with a texture analyser (TA, Stable Micro System, Surrey, UK). Typically, DCTs were put onto two aluminium nuggets fixed at a distance of 6.5 mm on the texture analyser. An aluminium probe was then set to move downwards at 1 mm/s against the DCTs. The maximum force to break each DCT from its centre was determined by force-distance plots using the Exponent™ Software (Stable Microsystems, Haslemere, UK). Mean values were calculated from 5 replicates for each drug reservoir formulation.

### 2.7 Drug disintegration and recovery tests

For each disintegration test, one drug tablet was placed in a glass vial containing 20 mL PBS solution and a small magnetic stirring bar. The solution was stirred at 1200 rpm using an IKA magnetic stirrer (IKA, Staufen, Germany). The time it took to completely dissolve the drug tablet was then recorded. The final dissolved drug solution was also used for drug recovery studies. Typically, they were further diluted with a dilution factor of 1000 using PBS solution. Finally, the diluted solutions were filtered and then analysed by the validated HPLC method to obtain the diluted drug concentration.

### 2.8 In vitro permeation study

*In vitro* permeation profile of the TC HCL HF-MAP was studied using a Franz cell setup. Neonatal porcine skin was used as the skin model. Skin samples were obtained from stillborn piglets and were trimmed to a thickness of 350 μm using an electric dermatome (Integra Life Sciences, Ratingen, Germany). As illustrated in Fig. 5 (a), the HF-MAP was first inserted manually into the skin and was then pressed firmly for 30 s. A 20 μL droplet of PBS solution was then dropped onto the back of the HF-MAP to facilitate the adhesion of the drug reservoir to the HF-MAP. Afterwards, the drug tablet (F4 & F9 in Table 1) was placed on the HF-MAP, after which a cylindrical stainless-steel weight was also placed on top of the drug reservoir and HF-MAP to ensure the HF-MAP was in place and remained inserted throughout the study. Finally, the entire donor compartment was mounted onto the receiver compartment and fixed by a clamp. The receiver compartment was filled with 12 mL of PBS release medium, which was maintained at 37 °C over the course of the study by circulating water (37 °C) through the water jacket.

At predetermined time points (30 min, 1 h, 2 h, 3 h, 4 h, 6 h, 24 h), 200 μL of the release medium in the receiver compartment was drawn from the sampling arm using a 1 mL syringe with a long stainless-steel needle. The same amount of release medium was then added back immediately to ensure the volume of release medium remain constant. The samples taken were then centrifuged at 12000 rpm for 15 min using an Eppendorf MiniSpin® centrifuge (Eppendorf UK limited, Stevenage, UK) and supernatants were injected onto the HPLC for analysing drug concentration. Dilutions were carried out where needed to ensure the drug concentrations were within the range of the validated HPLC method.

### 2.9 In vivo animal study

Prior to the study, approval was obtained from the Biological Services Unit (BSU) at Queen’s University Belfast, where the entire study was later carried out under Project License (PPL) 2794 by Personal License (PIL) holder 1892, 1991, 2136.

Female Sprague-Dawley rats were used in the *in vivo* animal study. In total, 24 animals were evenly separated in 4 groups, namely the control, oral gavage (OG), IV injection and MAP groups (*n* = 6 per group). The control group did not receive any drug. The OG, IV and MAP groups received tetracycline hydrochloride at a dose of 100 mg/kg, 50 mg/kg, 640 mg/kg, respectively.

The OG and IV injection procedures were performed by experienced staff at the BSU, whereas the MAP application was performed by PIL holders. On the day of MAP application, the hair on rats’ backs was removed to ensure maximum insertion of the MAPs. This was done by shaving the hair with an electric hair clipper followed by applying hair removal cream (Veet sensitive skin, Slough, UK) for 10 min. Prior to MAP application, rats were sedated using a gas anaesthesia procedure with isoflurane. Four PVP/PVA HF-MAPs were then manually inserted into the back of each rat using firm finger pressure. Twenty microlitre of water was dropped on the back of each MAP before applying one drug tablet onto the HF-MAP to facilitate the adhesion between the drug tablet and HF-MAP. A vapour permeable dressing film (Tegaderm™, 3 M) and 3 M kinesiology tape were subsequently used to wrap around the animals to ensure that the MAPs remain firmly inserted in the rats’ skin. The MAPs were kept in place for 24 h. Blood samples (approx. 200 μl each time) were drawn from the tail vein of each rat at pre-determined time points and were collected using heparinised Eppendorf tubes.

### 2.10 Extraction of rats’ plasma from whole blood

After blood samples were drawn from the rats, they were immediately centrifuged at 2000 *g* at 4 °C using a temperature-controlled centrifuge (Model: Sigma 2-16 K, Osterode am Harz, Germany). The supernatant plasma was then collected and stored in a freezer at –80 °C before further analysis. Where immediate processing of blood was not possible, the blood samples were temporarily stored in heparin containing Eppendorf tubes at 4 °C and were subsequently processed within 3 h.

### 2.11 Drug extraction from plasma

For each drug extraction, 100 μL of drug containing plasma was first aliquoted into a 1.5 μL Eppendorf tube. Afterwards, 300 μl of methanol was added into each plasma containing Eppendorf tube and vortexed for 1 h to allow for complete precipitation of plasma protein. Then the Eppendorf tubes were centrifugated at 3500 rpm for 10 min and the supernatant was collected and transferred into a glass tube, which was then placed in a TurboVap® LV evaporator (Zymark Corporation, Hopkinton, Massachusetts, USA) where all the liquid was subsequently evaporated. Finally, 100 μL H_2_O was added into the glass tube to reconstitute the drug solution, which was then injected into the HPLC for drug quantification.

### 2.12 Pharmaceutical analysis

Quantification of tetracycline was performed by reverse-phase high-performance liquid chromatography (RP-HPLC) using an Agilent 1200 series HPLC system throughout this work. Agilent ChemStation® software was used for chromatogram analysis. The mobile phase consisted of 80% of 0.02 M potassium phosphate monobasic (adjusted to pH 2.5 with 85% phosphoric acid) and 20% of acetonitrile. Flow rate, column temperature and run time were set at 0.3 mL/min, 25 °C, and 9 min, respectively. Injection volume was 20 μL, whilst UV detection was set at 270 nm. A Luna® Omega 3 μm PS C18 100 Å 150 × 3.0 mm column from Phenomenex (Torrance, California, USA) was used for this method. For analysis of plasma samples, the same chromatographic conditions were used. However, a Phenomenex SecurityGuard™ column (Torrance, California, USA) was connected to the front of the Luna® column to protect it from excessive biological impurities, which may otherwise damage the column. All samples were either centrifuged or filtered before being injected onto the HPLC. Both *in vitro* and *in vivo* HPLC methods were validated in accordance with the International Council on Harmonization (ICH) guidelines [32].

### 2.13 Statistical analysis

Least squares linear regression analysis, correlation analysis and calculation of means and SDs were all carried out using Microsoft Excel 2016 (Microsoft Corporation, Redmond, USA). An unpaired *t*-test was used for comparison of two groups and a one-way analysis of variance (ANOVA) with Tukey’s post-hoc test was used when comparing three or more groups. In all cases, *p* < 0.05 was considered statistically significant. All statistical analyses were performed by GraphPad Prism 5 (GraphPad Software Inc. San Diego, California, USA).

## 3 Results and discussion

**Fig. 1:**
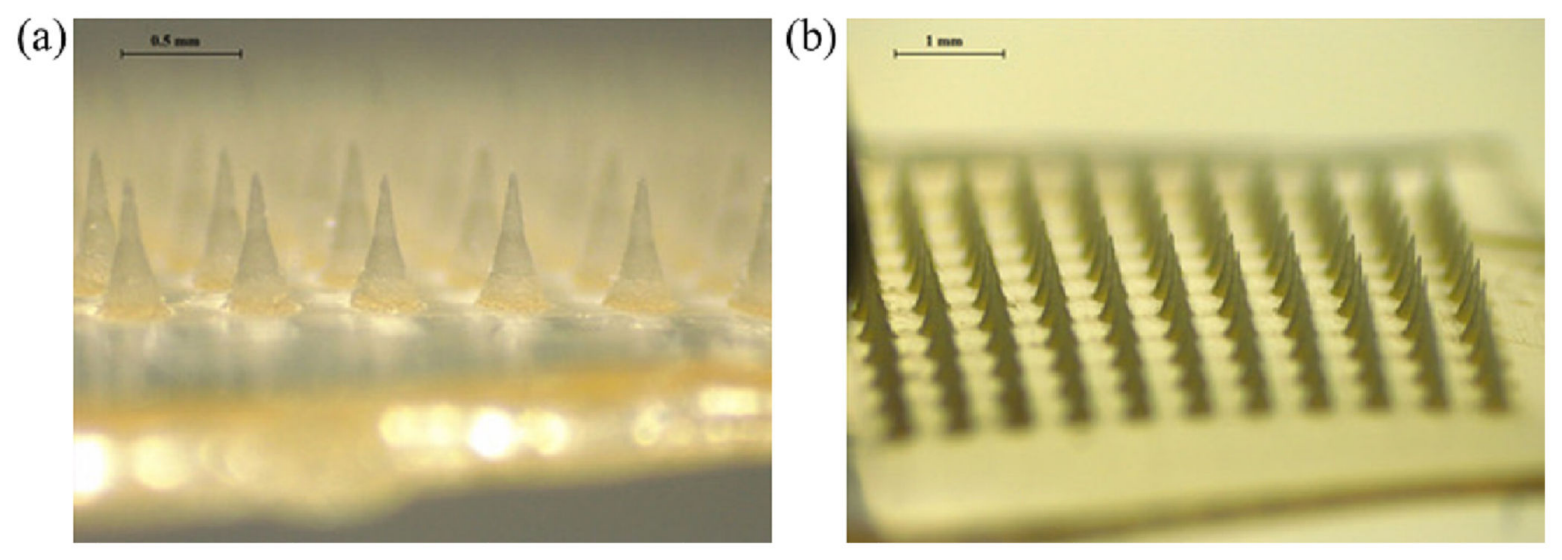
Microscopic images of PVP/PVA HF-MAP.

Figure 1 (a) and (b) are the representative microscopic images of the resulting PVP/PVA HF-MAP, which seems sharp in the tips.

**Fig. 2:**
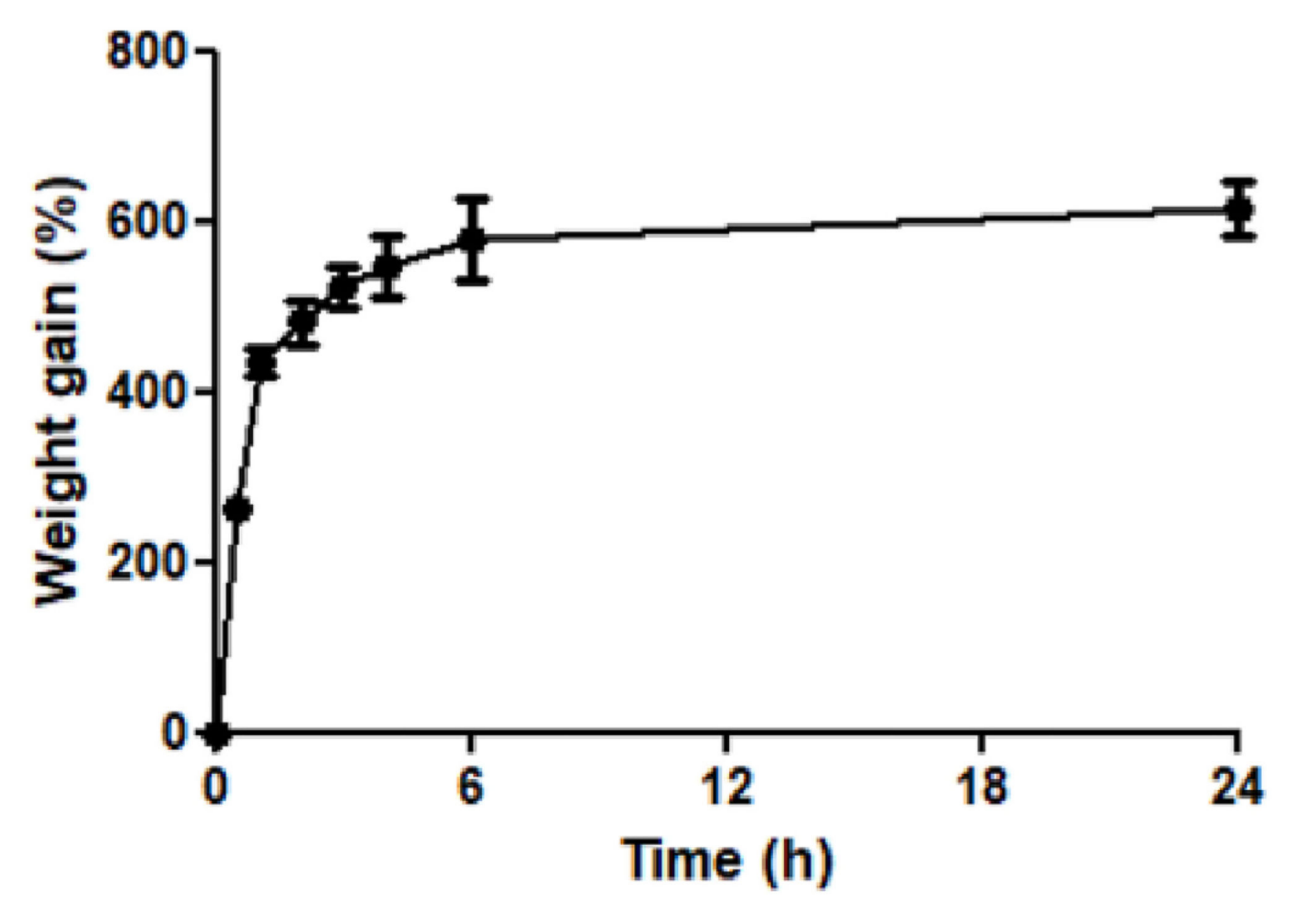
Weight gain of PVP/PVA films in PBS buffer (pH 7.4) over 24 h (*n* = 6).

The mechanism of the HF-MAP drug delivery system sees the HF-MAP take up interstitial fluid from the skin, into which the drug reservoir dissolves and permeates into the skin. As a result, one of the key factors in deciding the success of such a drug delivery system is the ability of HF-MAP to absorb aqueous media within a short period of time. Therefore, a swelling test was first done for the PVP/PVA HF-MAP, which has been described above. As shown in Fig. 2, a rapid swelling of the PVP/PVA HF-MAP was observed during the first hour, with a weight gain of over 400%. The PVP/PVA HF-MAPs kept swelling at a slower rate until 6 h, reaching a weight gain of just under 600%. Afterwards, no significant further swelling was observed. The final weight gain at 24 h was 614.85 ± 33.26%.

These results showed that the PVP/PVA HF-MAP is capable of taking up aqueous fluid at a rapid rate, which is ideal for delivering hydrophilic drugs. When used to deliver drugs transdermally, the HF-MAP needs to take up interstitial fluid upon insertion into skin in order to allow the drugs to be dissolved and permeate into skin. As a result, the quick swelling capability of PVP/PVA HF-MAP would reduce the time it takes for the drug to enter the blood circulation.

**Fig. 3:**
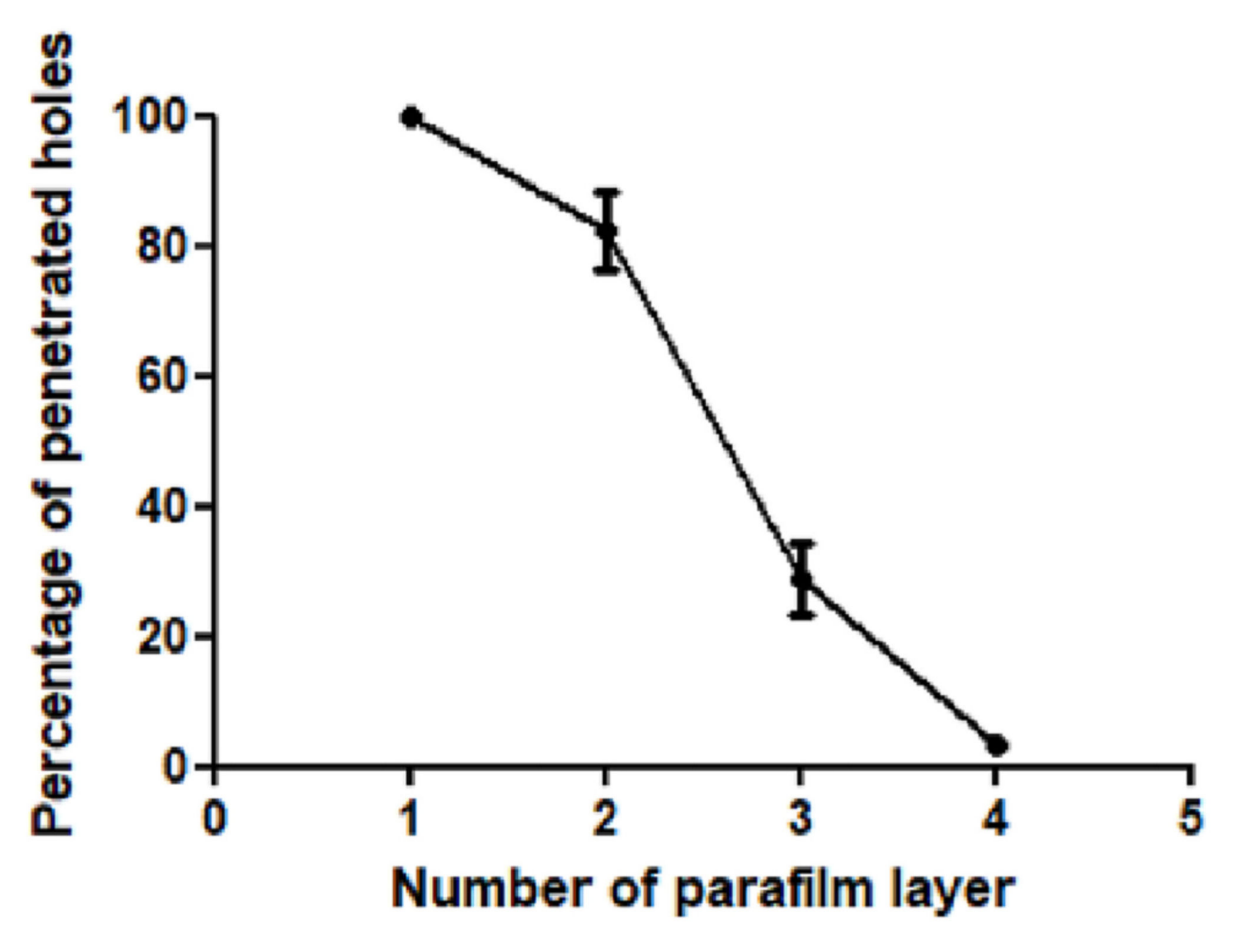
Percentage of holes penetrated by PVP/PVA HF-MAP on each Parafilm layer (means ± S.D., *n* = 3, *p* < 0.0001).

**Fig. 4:**
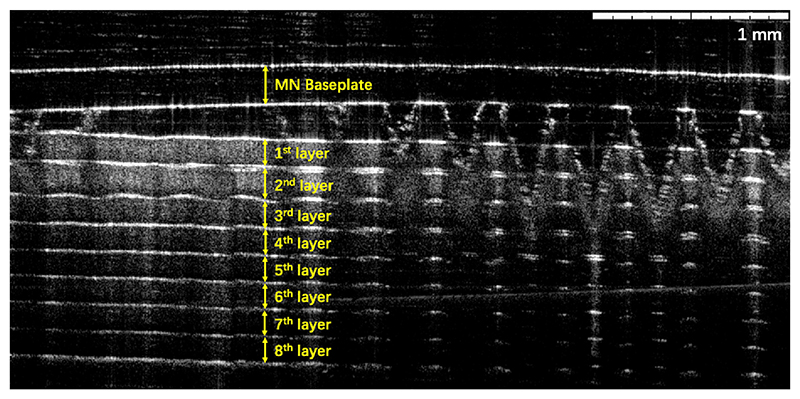
OCT image of PVP/PVA HF-MAP inserted into Parafilm using Texture Analyser.

Successful insertion of MAPs into skin bypassing the *stratum corneum* is a key step to ensure successful drug delivery, as fully inserted MAPs would allow for more interstitial fluid to be taken up and subsequently more drug to permeate into skin. A variety of different approaches have been developed to characterise MAP insertion [33–36]. In the present study, the insertion capability of the PVP/PVA HF-MAP into a skin simulant, eight-layer Parafilm M, which has been widely used as a standard model for testing skin insertion capability of MAPs due to its simplicity and low cost [37,38]. The percentage of MN insertion for each layer has been illustrated on Fig. 3. A complete penetration of MNs into the first Parafilm layer was achieved with 100% penetration across all replicates. In comparison, 82.3 ± 6.1% and 24.6 ± 5.5% of MNs penetrated the second and third Parafilm layer, respectively. The fourth parafilm layer was only penetrated by 3.1 ± 1.4% of MNs. This test is used to estimate MAP in a simple way without using biological tissue. According to Larrañeta et al. that developed this test insertion depth can be established between the last layer that contains >20% of holes and the following one [37]. In this case the insertion depth will range between the 3rd layer (378 μm) and the 4th layer (504 μm). An OCT image in Fig. 4 can further prove the successful insertion of MNs into Parafilm. It can be clearly seen that the PVP/PVA HF-MAP can penetrate 3–4 layers of Parafilm. Since the thickness of human *stratum corneum* ranges from 12 μm in the back to 40 μm in the abdomen, whilst one parafilm layer is 126 μm in thickness, the complete insertion of MNs across first Parafilm layer, demonstrated that full insertion of MNs across the *stratum corneum* can be reasonably expected [39–42].

In total, 9 different formulations of drug reservoir were studied and underwent subsequent characterisation. The direct compression technique was used to manufacture these solid drug reservoirs. This is because it is a straightforward process that is less time consuming and more energy efficient than other formulation techniques, such as lyophilised drug tablets and drug containing polymeric films. All drug reservoir formulations contained the active pharmaceutical ingredient TC HCL and two extensively-used pharmaceutical excipients, namely mannitol and CCS, contained in a number of approved pharmaceutical products and which have successfully been used to prepare directly compressed drug reservoirs for HF-MAP based transdermal drug delivery previously [31]. In particular, mannitol has been increasingly used as a filler/binder for solid dosage forms and CCS has predominantly been used as a disintegrant [43,44]. The use of these two excipients offers an excellent balance between the integrity and robustness of drug tablets and their prompt disintegration upon contact with aqueous fluid. Table 1 summarises the list of drug reservoir formulations. The drug content ranged from 70 to 90% with varying percentages of mannitol and CCS used. Three characterisations, including a mechanical test, drug recovery and drug tablet disintegration were then carried out. The mechanical test looked at the extent to which the drug reservoir is capable of resisting an external break force. This is particularly important for the drug tablets, as they may undergo various harsh environments from the time of manufacturing through various transportation processes until reaching the end user. Therefore, the drug reservoir must remain intact and mechanically robust at the time of use. Consequently, a fracturability test, which has commonly been used in the food industry, was used to test the mechanical properties of the resulting drug reservoirs [45]. It aimed to provide an indication of whether the drug reservoir would crumble when it is applied on skin. Table 1 summarises the break force for each drug reservoir. F1-F3 were quite brittle, and they were broken by relatively low break forces, indicating their mechanical strengths were not satisfactory. This might be attributed to the binder (<10%) being not enough to maintain the physical strength of the drug tablets. Similarly, F6 which contained only 5% mannitol was brittle too. All other formulations were easy to handle, without risk of being broken easily.

Drug recovery is also an important factor in drug formulation because low drug recovery results in higher levels of drug waste. Low drug recovery also increases the requirement for a bulkier drug reservoir in order to deliver a certain amount of drug if drugs cannot be effectively recovered from the reservoir. It can be seen from Table 1 that all formulations have over 95% drug recovery, which is comparable with previous studies [31]. The lost drug might have been incorporated within the insoluble CSS and been filtered out before drug quantification.

*In vitro* drug disintegration tests were also performed to characterise the disintegration characteristics and dissolvability of the drug reservoirs. Table 1 also summarises the disintegration time of the drug reservoir formulations. The three formulations with 90% drug content (F1-F3) dissolved within 30 s, possibly due to the lack of binders in their formulations. Other formulations that contained a higher percentage of CCS tended to dissolve faster as well. All formulations could dissolve within 90 s. As demonstrated in previous research, a disintegration time of <5 min in release medium is generally preferred for drug reservoirs used in MAP-based drug delivery [31].

Ideally, drug reservoirs should be mechanically robust and also able to disintegrate in aqueous medium quickly. F1, F2, F3 and F6 tend to be brittle (*p* = 0.3207 for break force), whereas F5, F7, F8 disintegrate more slowly, than other formulations. As a result, F4 and F9 were selected for *in vitro* release studies as they were mechanically robust (with no significant difference, *p* = 0.4928) whilst also able to dissolve quickly in aqueous medium. There was no significant difference in their percentage drug recoveries (*p* = 0.2457).

HPLC methods for the analysis of all drugs containing samples including *in vivo* plasma samples were validated in accordance with the ICH guidelines [32]. Table 2 summarises the slope, y-intercept, correlation coefficient as well as limits of detection and limits of quantification.

**Fig. 5:**
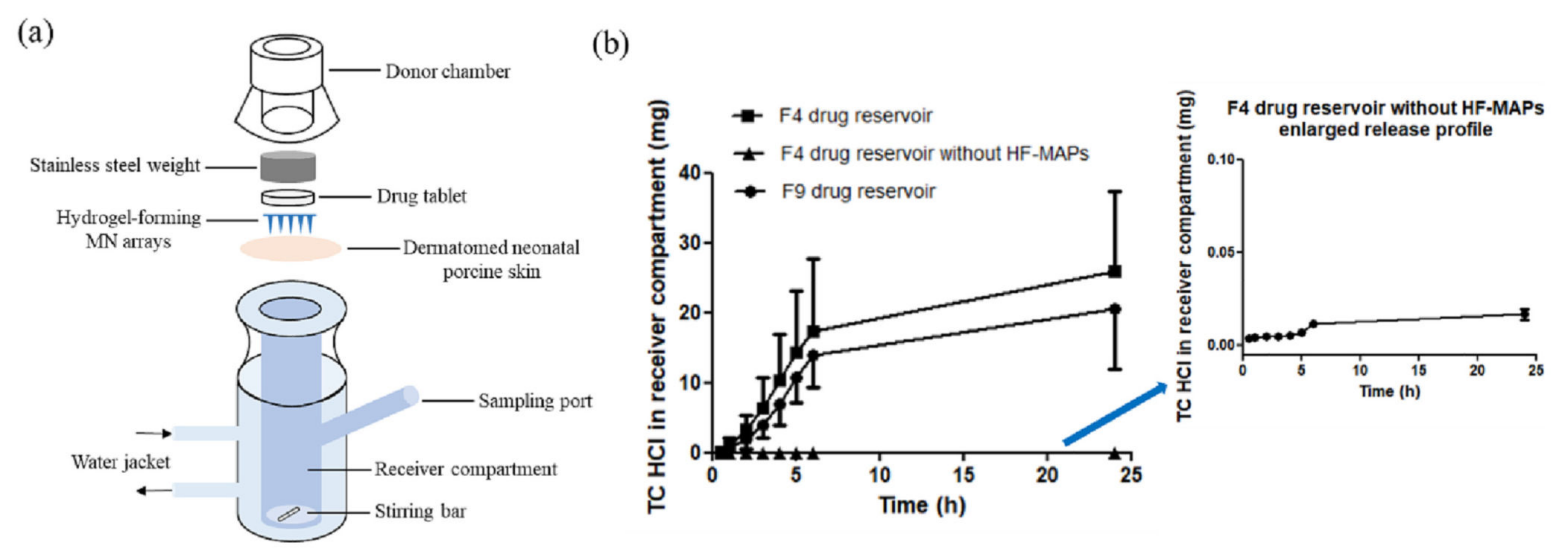
(a) Franz cell setup for conducting the *in vitro* drug permeation studies and (b) *In vitro* drug permeation profile of F4 and F9 drug reservoirs using PVP/PVA HF-MAP and dermatomed porcine skin (Means ± S.D., *n* = 4).

The drug permeation study was performed using the apparatus as illustrated in Fig. 5 (a). The Franz cell apparatus has been used as a standard *in vitro* permeation apparatus in MAP-based transdermal drug delivery research [46,47]. Fig. 5 (b) shows the drug permeation kinetics of F4 and F9 over the course of 24 h.

Drug tablets for both F4 and F9 weighed 150 mg, containing 120 mg and 105 mg of API, respectively. It can be seen from Fig. 5 (b) that drug permeation from both F4 and F9 reservoir seemed to lag in the first 2 h, especially during the first 0.5 h, where only 0.21 ± 0.12 mg and 0.13 ± 0.08 mg of drug were permeated from F4 and F9 drug reservoirs, respectively. This can be likely attributed to the time taken for release medium to reach and dissolve the drug. Upon mounting the donor compartment onto the receiver compartment, the HF-MAP tips inserted across the dermatomed porcine skin would take up the release medium in the receiver compartment, which would then diffuse across the hydrogel network within the HF-MAP, resulting in a swollen HF-MAP. Simultaneously, the fluid in contact with the drug reservoir would start to dissolve the drug reservoir at the back of HF-MAP. Therefore, there would be some degree of delay in drug permeation into the receiver compartment initially due to the time for the release medium to diffuse across the hydrogel network and the drug to be dissolved and permeate through the swollen HF-MAP. The dissolved drug from both formulations then steadily permeated across the HF-MAPs at a relatively stable speed until 6 h, where 17.26 ± 10.38 mg and 13.95 ± 4.68 mg of TC HCL had permeated into the receiver compartment. Drug permeation gradually slowed down in the remainder of the measured time. By the end of the permeation study at 24 h, in total 25.94 ± 11.40 mg and 20.57 ± 8.56 mg of drug were found in the receiver compartment for F4 and F9, equivalent to 21.6 ± 9.5% and 19.6 ± 8.2% of drug delivery, respectively. These results are in line with previously reported delivery efficiencies for hydrogel-forming MAPs [21]. The delivery efficiency depends highly on the type of drug selected. Moreover, these results are in line with the delivery data reported for different types of conventional transdermal patches that can deliver as little as 5% of the initial drug loaded in the patch [48]. Whilst the HF-MAP created an increased diffusion path upon swelling, the majority of drug content had been dissolved and released into the receiver compartment by 6 h. This is likely to explain the decreased drug permeation rate after 6 h. This is expected as drug release is limited by the partition of drug across different interfaces. In order to permeate through the skin, TC HCL should dissolve first in fluid. Subsequently, the drug in solution should permeate inside the hydrogel matrix and finally it should diffuse from the hydrogel into the skin. These processes are governed by concentration gradients and in the closed Franz cell system the concentration gradient between the MAP hydrogel matrix and the reservoir compartment will decrease with time. This explains why drug permeation is slower at longer times.

In addition, another set of F4 was also applied on porcine skin on Franz cells without using HF-MAPs. It can be clearly seen from the drug permeation data drug permeation was limited. <0.025 mg of drug permeated across the skin. Therefore it can be established that the use of HF-MAPs increased drug permeation by a factor of 1000. These results are not surprising considering that previous studies showed that TC HCL presented limiting permeability across the skin [49].

From the drug permeation kinetics of both formulations, it is apparent that the overall release kinetics of F4 and F9 are similar over the course of 24 h, with no significant difference in the amount of drug delivered from each formulation (*p* = 0.5878). The *in vitro* permeation study proved that both F4 and F9 drug reservoirs dissolved well along-side PVP/PVA HF-MAP in an integrated drug delivery system. F4 was selected for *in vivo* animal studies as it contained more drug in each drug reservoir.

It is important to note that during this study MAPs and reservoirs have been assembled just before the experiments. However, we think that both parts should be manufactured and assembled before packaging if this product is intended for patient’s use. Complex application process can lead to patient compliance issues [50]. Therefore, MAP devices should aim to be easy to apply.

In the *in vivo* study, each rat in the MAP treatment group received four TC HCL MAPs (equivalent to 640 mg/kg), whereas each rat in OG and IV groups received the drug at a dose of 100 mg/kg and 50 mg/kg, respectively. Dose selection in the MAP treatment group was based on the relatively low bioavailability of transdermal drug delivery systems, even with the enhancement offered by HF-MAP [31]. As part of an exploratory study, it was also important to determine how much drug could be delivered *in vivo* and whether this system could constitute a sufficient dose of drug. Similar doses have already been shown to be safe to rats in previous research [51,52]. The pharmacokinetic parameters of the three treatment groups are presented in Table 3, which were calculated using PKSolver software.

**Fig. 6:**
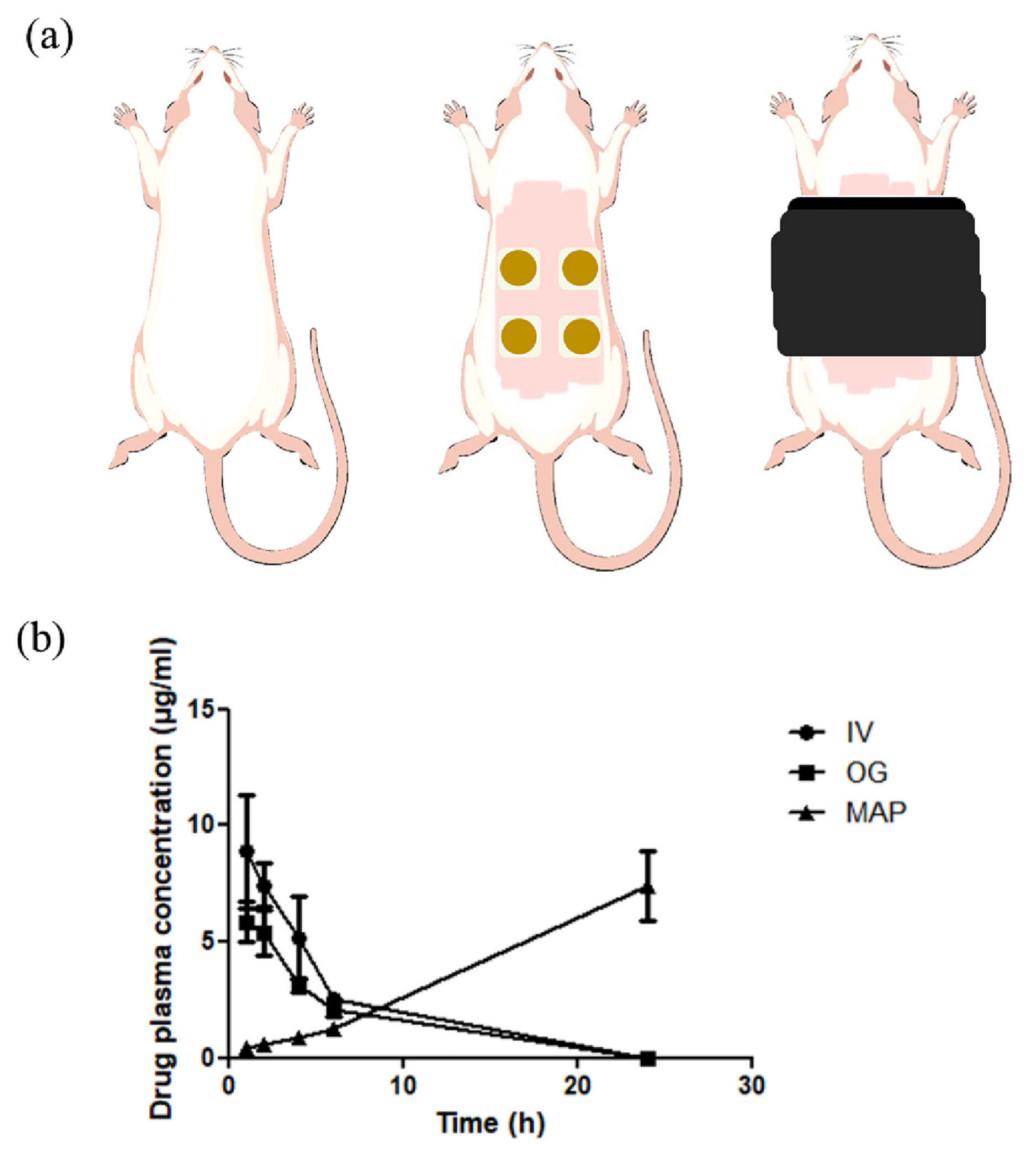
(a) Illustration of *in vivo* pharmacokinetics study using rats, rats as received (left), rats after HF-MAP application (middle), Rats after HF-MAP application with tape wrapping (right) and (b) *In vivo* pharmacokinetics of TC HCL by three routes of administration (IV, OG, and MN) (*n* = 3 at 1, 2, 4, 6 h and *n* = 6 at 24 h).

It can be seen in Fig. 6 (b) that TC HCL was quickly absorbed by rats in both OG and IV groups. For the oral group, the drug was first detected in plasma at 5.86 ± 1.48 μg/ml at 1 h post administration, which was also the peak plasma concentration. Afterwards, the drug plasma concentration quickly decreased over the next few hours and was detected at 2.06 ± 0.51 μg/ml at 6 h. There was no detectable TC HCL remaining in plasma at 24 h. The IV group showed similar pharmacokinetics. Drug plasma concentration peaked at 8.86 ± 4.19 μg/ml 1 h post drug administration. It then decreased over the next few hours, with 2.95 ± 2.22 μg/ml of drug being detected in plasma at 6 h. Similarly, no remaining drug was detected in plasma at 24 h.

The pharmacokinetics of the MAP group showed a very different pattern in drug plasma concentration over the course of 24 h. Initially, only 0.43 ± 0.11 μg/ml of TC HCL was detected in plasma at 1 h post MAP application. It then steadily increased over time until 24 h. The drug plasma concentrations at 2, 4, 6, 24 h were 0.58 ± 0.30 μg/ml, 0.84 ± 0.23 μg/ml, 1.24 ± 0.35 μg/ml, 7.40 ± 4.74 μg/ml, respectively.

It is assumed that all drugs are absorbed by rats in the IV group. Therefore, the transdermal and oral bioavailability can be calculated using Eq. 3.

(3) $$\frac{\text{AUC}(\text{MAP}\;\text{or}\;\text{Oral})}{\text{AUC}(\text{IV})}=\frac{F(MAP\;or\;Oral)Dose(MAP\;or\;Oral)}{Dose(IV)}$$

where F(MAP or Oral) is the transdermal or oral bioavailability.

With the ratio between AUCMAP and AUCIV presented in Table 2, it can be known that approximately 30.55 mg of drug was absorbed by each rat in the MAP group, representing a 19.1% transdermal bioavailability of the TC HCL HF-MAP system. The oral bioavailability is 33.5%, which was calculated using the same formula. Despite being a relatively high delivery efficiency for a transdermal drug delivery system, it is still important to highlight that transdermal MAP bioavailability may still have been underestimated as the drug plasma concentration of the MAP group was still increasing at 24 h. A similar phenomenon was also observed in *in vivo* studies of three different MAPs in a previous study [31]. A probable explanation is that despite complete disintegration of drug reservoir, the HF-MAPs still contained a lot of drug when they were removed at 24 h. Accordingly, the release of drug from the hydrogel had not completed as we can see that the drug plasma concentration had not peaked upon removal of MAPs. The sustained drug delivery could last for an even longer time if the MAPs were left on the site of application. However, due to animal welfare regulations at the BSU, the maximum duration of MAP application permitted is 24 h. The current pharmacokinetics reflect a real world scenario where repeated dosing of antibiotics is needed to treat infections either by oral administration or IV injection due to rapid antibiotic elimination from the body. In comparison, transdermal delivery of antibiotics using HF-MAP could potentially reduce the frequency of antibiotic administration by providing sustained release.

## 4 Conclusion

A novel antibiotic HF-MAP system has successfully been designed, fabricated, and evaluated in the present work. *In vivo* pharmacokinetics studies using a Sprague Dawley rat model showed sustained release of TC HCL was achieved by HF MAP-based transdermal delivery, in comparison to OG and IV administration. This offers a unique opportunity to allow users to self-apply antibiotic MAP products, which deliver antibiotic systemically with reduced dosing frequency. A comprehensive dysbiosis study is currently being carried out, which may uncover the extent to which antibiotic MAP system effect gut microbial populations compared with OG and IV antibiotic administration. From a commercial standpoint, MAP-based pharmaceutical products may prove less economical than conventional oral products in the short term. However, the cost of antibiotic MAP products may gradually be brought down as large-scale manufacturing of MAPs becomes widely feasible. Reducing cost of production, alongside the advantages over IV administration and current predictions regarding AR emergence, is likely to encourage health care providers to consider their widespread implementation in future. Increased collaboration among healthcare providers, pharmaceutical companies, public health agencies, and governments is needed to help realise the potential this innovative delivery system has to offer.

## Figures and Tables

**Table 1:** Composition and characterisation of directly compressed tetracycline tablets.

Formulation	Compositions				Tablet Characteristics		
	TC HCL (%)	Mannitol (%)	CCS (%)		Break force (N) Means ± S.D. *n* = 5	Drug recovery (%) Means ± S.D. *n* = 3	Disintegration time (s)
F1	90	5	5		3.18 ± 0.44	99.24 ± 1.62	<30
F2	90	7.5	2.5		3.02 ± 0.52	98.71 ± 2.95	<30
F3	90	2.5	7.5		2.89 ± 0.36	99.53 ± 1.48	<30
F4	80	10	10		5.25 ± 0.57	98.53 ± 2.63	<60
F5	80	15	5		5.11 ± 0.30	97.51 ± 3.05	<90
F6	80	5	15		3.57 ± 0.43	97.28 ± 2.38	<30
F7	70	15	15		5.64 ± 0.72	96.12 ± 2.87	<90
F8	70	20	10		5.81 ± 0.57	97.39 ± 1.85	<90
F9	70	10	20		5.55 ± 0.39	95.75 ± 2.46	<60

**Table 2:** Parameters of calibration curves for *in vitro* and *in vivo* TC HCL quantification.

HPLC method	Slope	y-intercept	R^2^	LoD (μg/mL)	LoQ(μg/mL)
*In vitro* method	133.08	3.34	1	0.03	0.10
*In vivo* method	156.22	17.96	0.9996	0.15	0.45

**Table 3:** Pharmacokinetic parameters of OG, IV, and MAP groups (means ± SD, *n* = 3 at 1, 2, 4, 6 h and *n* = 6 at 24 h).

Parameter	Oral	IV	MAP
Dose (mg/kg)	100	50	640
AUC (μg*h/mL)	22.48	33.56	82.03
Tmax (h)	1	1	24 (as of 24 h)
Cmax (μg/mL)	5.86 ± 1.48	8.86 ± 4.19	7.40 ± 4.74 (as of 24 h)
t1/2 (h)	2.88	2.84	

## Data Availability

Data will be made available on request.
